# Follicular T Cells from smB^−^ Common Variable Immunodeficiency Patients Are Skewed Toward a Th1 Phenotype

**DOI:** 10.3389/fimmu.2017.00174

**Published:** 2017-02-27

**Authors:** Vanesa Cunill, Antonio Clemente, Nallibe Lanio, Carla Barceló, Valero Andreu, Jaume Pons, Joana M. Ferrer

**Affiliations:** ^1^Immunology Department, Hospital Universitari Son Espases, Palma de Mallorca, Balearic Islands, Spain; ^2^Human Immunopathology Research Laboratory, Institut d’Investigació Sanitària de Palma (IdISPa), Palma de Mallorca, Balearic Islands, Spain

**Keywords:** B cells, CVID, follicular T helper cells, regulatory follicular T cells, Th17.1 cells

## Abstract

Germinal center follicular T helper (GCTfh) cells are essential players in the differentiation of B cells. Circulating follicular T helper (cTfh) cells share phenotypic and functional properties with GCTfh cells. Distinct subpopulations of cTfh with different helper capabilities toward B cells can be identified: cTfh1 (CXCR3^+^CCR6^−^), cTfh2 (CXCR3^−^CCR6^−^), and cTfh17 (CXCR3^−^CCR6^+^). Alterations in cTfh function and/or distribution have been associated with autoimmunity, infectious diseases, and more recently, with several monogenic immunodeficiencies. Common variable immunodeficiency (CVID) disease is the commonest symptomatic primary immunodeficiency with a genetic cause identified in only 2–10% of patients. Although a heterogeneous disease, most patients show a characteristic defective B cell differentiation into memory B cells or antibody-secreting cells. We investigated if alterations in CVID cTfh cells frequency or distribution into cTfh1, cTfh2, and cTfh17 subpopulations and regulatory follicular T (Tfr) cells could be related to defects in CVID B cells. We found increased percentages of cTfh exhibiting higher programmed death-1 expression and altered subpopulations distribution in smB^−^ CVID patients. In contrast to smB^+^ patients and controls, cTfh from smB^−^ CVID patients show increased cTfh1 and decreased cTfh17 subpopulation percentages and increased CXCR3^+^CCR6^+^ cTfh, a population analogous to the recently described pathogenic Th17.1. Moreover, Tfr cells are remarkably decreased only in smB^−^ CVID patients. In conclusion, increased cTfh17.1 and cTfh1/cTfh17 ratio in CVID patients could influence B cell fate in smB^−^ CVID patients, with a more compromised B cell compartment, and the decrease in Tfr cells may lead to high risk of autoimmune conditions in CVID patients.

## Introduction

Common variable immunodeficiency (CVID), the most common symptomatic primary antibody disorder in adults, is an enigmatic primary immunodeficiency (PID) characterized by hypogammaglobulinemia, absent or impaired specific antibody production, and immune dysregulation. Patients suffer from respiratory and/or gut recurrent infections, although chronic gastroenteropathies, autoimmunity, and even malignancy, are also often present.

Patients benefit from substitutive gammaglobulin therapy (1–3). Genetic mutations and polymorphisms account only for 2% of CVID patients while for most of them, the underlying molecular defect remains unknown (4). Both T and B cells dysfunctions have been described in CVID patients. In spite of its heterogeneity, the most common defect to all patients is a failure of final B cell differentiation into memory B cells or antibody-secreting cells. Accordingly, patients have been classified depending on their number of naïve, non-switched and switched-memory B cells (5–7) in order to more accurately address the relationship between experimental findings and CVID phenotypes. For instance, a low percentage of memory B cells in CVID patients has been associated with a worse clinical presentation and poor response to vaccines (7, 8).

Protective immunity after infection or vaccination relies on the production of effective antibodies by B lymphocytes. Follicular helper T (Tfh) cells have been identified in the recent years as a CD4^+^ T cell subpopulation specialized in directing the maturation and differentiation of B cells in the germinal centers. Tfh cells were first described as a CXCR5 expressing population localizing in tonsillar follicles and supporting B cell maturation and immunoglobulin production (9, 10). CD4^+^CXCR5^+^ follicular T cells also express ICOS, programmed death (PD)-1, and secrete high levels of interleukin (IL)-21. The most important transcription factor for the generation of Tfh cells is Bcl-6 (11–13). The difficulty to access to secondary lymphoid organs has hampered the study of Tfh cells in humans. The discovery of a circulating counterpart of this population has allowed investigating their relevance in health and disease. Although controversy still exists, this circulating Tfh (cTfh) population is characterized by a CD4^+^CXCR5^+^ phenotype, variable expression of CD40L, ICOS, PD-1, Bcl-6, CCR7, and IL-21 secretion. Heterogeneity exists among cTfh population. According to the expression of CXCR3 and CCR6, they can be classified into cTfh1 (CXCR3^+^CCR6^−^), cTfh2 (CXCR3^−^CCR6^−^), and cTfh17 (CXCR3^−^CCR6^+^) that produce a different set of cytokines and whose differentiation relies on distinct transcription factors (14). Tfh2 and Tfh17, but not Tfh1, cells are able to help naïve B cells (14, 15).

Follicular helper T cells play a role in the pathogenesis of several diseases. Circulating Tfh cells are increased in a group of autoimmune diseases characterized by autoantibody production-like systemic lupus erythematosus (16, 17), Type I diabetes mellitus (18), Sjögren syndrome (19), juvenile dermatomyositis (14), Guillain–Barré syndrome (20), or multiple sclerosis (21) among others.

The study of monogenic PIDs has evidenced a Tfh cells decrease in patients with hyper-IgM syndrome caused by mutations in CD40L, ICOS-deficient patients (22), and XLA patients (23), all of them characterized by defects in B cell maturation and immunoglobulins production. Patients with hyper-IgE syndrome caused by STAT3 mutations show impaired antibody responses and are also deficient in circulating CXCR5^+^CD4^+^ cells (24). Moreover, not only the quantity, but also the quality of Tfh cells is altered in monogenic PIDs (25).

Another important evidence of the role of Tfh cells in antibody production comes from the study of HIV patients. Although there is an expansion of Tfh cells in HIV infected individuals, impaired generation of neutralizing antibodies is related to deficient Tfh help to B cells (15) whereas high frequency of CD4^+^PD1^low^CXCR3^−^CXCR5^+^ is related to broadly neutralizing antibodies to HIV (26). However, due to the differences in the selection of the patients sample and in the markers used to identify cTfh cells, controversy still exists.

The aim of this study was to evaluate cTfh cells and their subpopulations in CVID patients and relate the results to the distribution of memory B cell subpopulations.

## Materials and Methods

### Patients

CVID patients (*n* = 34) were selected according to diagnostic criteria of the European Society for Immunodeficiencies scientific group (27). Patients were classified into two groups according to the European consensus classification for CVID (EUROclass) (7) as (i) CVID patients with ≤2% of IgD^−^CD27^+^ (switched memory phenotype) B cells or smB^−^, and (ii) patients with >2% of IgD^−^CD27^+^ B cells or smB^+^. Patients with less than 1% B cells were excluded.

None of our studied patients had trauma, infections or received systemic steroids, standard immunosuppressive therapies, or biologic therapies at least the 3 months previous to the time of study. CVID patients received intravenous gammaglobulin therapy every 21–28 days. Peripheral blood samples were collected before gammaglobulin replacement. Patient 23 was recruited at the time of diagnosis and he had not begun the treatment. Table 1 summarizes the patients’ age, gender, percentages of B cell subpopulations, autoimmune manifestations, and the presence of enteropathy. Table S1 in Supplementary Material depicts extended T CD4^+^ cell immunophenotype of CVID patients. Age- and sex-matched healthy blood donors (*n* = 37) were included as controls. The study was conducted according to the ethical guidelines of the 1975 Declaration of Helsinki and approved by CEIC (Balearic Islands Clinical Research Ethics Committee; IB 2517/15) and written informed consent was obtained from all subjects.

**Table 1:** **Age, gender, immunoglobulin levels, B-cells subpopulations, autoimmune manifestations, and enteropathy presence of CVID patients**.

Patient	Age (years)	Age at diagnosis (years)	Sex (male/female)	IgG (mg/dl)	IgA (mg/dl)	IgM (mg/dl)	CD19 (%)	CD19^+^ (%)				EUROclass group	Autoimmune manifestations	Enteropathy
								CD21 (low)	IgD^+^CD27^−^	IgD^+^CD27^+^	IgD^−^CD27^+^			
1	65	55	M	316	9	12	6	19	91	7	<1	smB^−^	Hemolytic anemia	–
2	33	20	M	82	<6	5	10	11	94	3	<2	smB^−^	–	–
3	33	21	F	351	20	<6	15	24	81	11	2	smB^−^	–	–
4	64	49	F	112	<6	<5	6	16	87	7	<1	smB^−^	–	–
5	64	52	M	77	<6	<4	2	26	86	7	<2	smB^−^	–	–
6	71	66	F	37	<6	<5	4	33	82	5	<1	smB^−^	–	–
7	42	36	M	85	<24	<18	6	13	89	7	<1	smB^−^	Lichen planus	–
8	31	25	F	330	107	96	12	10	92	5	<1	smB^−^	Thrombopenia	–
9	83	68	F	481	<7	52	16	16	84	5	2	smB^−^	–	–
10	33	30	F	164	<7	8	24	5	89	6	<2	smB^−^	–	^+^
11	77	67	F	323	98	<6	24	55	73	26	<1	smB^−^	–	–
12	27	22	M	316	<25	21	14	35	91	4	<2	smB^−^	–	–
13	52	46	F	397	126	22	16	5	83	15	<1	smB^−^	–	–
14	36	28	M	75	<25	<17	12	34	82	10	<2	smB^−^	–	^+^
15	32	16	M	461	<28	<17	17	17	87	7	<1	smB^−^	–	^+^
16	40	25	M	382	<6	13	2	NA	86	12	2	smB^−^	Thrombopenia, neutropenia	–
17	33	15	M	229	<6	12	6	66	91	1	1	smB^−^	Hemolytic anemia, thrombopenia	^+^
18	67	57	M	452	40	30	4	69	93	3	<1	smB^−^	–	–
19	29	14	F	<7	<7	<5	5	67	79	16	<1	smB^−^	–	–
20	33	30	F	33	7	4	2	44	93	4	<1	smB^−^	–	^+^
21	45	40	F	772^a^	<7^a^	<5^a^	7	41	97	1	<1	smB^−^	–	–
22	42	29	F	7	<6	<4	2	NA	99	1	0	smB^−^	Neutropenia	–
23	28	28	M	13	<7	<5	5	4	93	5	1	smB^−^	–	^+^
24	71	58	M	445	<23	<17	16	6	76	15	5	smB^+^	Neutropenia, Cogan syndrome	–
25	48	36	F	288	32	14	20	6	55	13	17	smB^+^	–	^+^
26	51	36	F	495	44	38	11	7	50	9	15	smB^+^	–	–
27	37	29	F	578	<25	62	9	20	84	8	4	smB^+^	–	–
28	86	72	F	253	26	<16	2	20	39	14	36	smB^+^	Vitiligo	–
29	71	66	F	434	47	46	14	20	64	25	4	smB^+^	–	–
30	15	13	F	471	<7	10	14	11	63	32	3	smB^+^	–	–
31	65	50	F	327	73	30	20	5	70	15	9	smB^+^	Cutaneous lupus erythematosus	^+^
32	71	55	F	452	46	58	7	5	47	13	19	smB^+^	–	–
33	44	40	F	387	<7	17	6	8	70	11	10	smB^+^	–	–
34	63	42	F	480	<6	39	14	6	77	19	2	smB^+^	–	–

*Current age (years), age at diagnosis (years), gender (M, male; F, female), seric immunoglobulin levels (IgG, IgA, and IgM) before starting replacement therapy, percentage of peripheral blood B-cells (CD19^+^), percentages of CD21^low^, naïve (IgD^+^CD27^−^), unswitched memory (IgD^+^CD27^+^), and switched memory (IgD^−^CD27^+^) B-cells subpopulations (referred to total CD19^+^ B-cells), EUROclass classification, autoimmune manifestations, and enteropathy presence of CVID patients*.

*NA, not available*.

*^a^Values after replacement therapy*.

### Isolation of Peripheral Blood Mononuclear Cells (PBMCs), T Cell Sorting, and Cell Culture

Peripheral blood mononuclear cells were isolated from heparinized blood by Ficoll density gradient centrifugation. PBMCs were resuspended in culture medium: RPMI-1640 supplemented with 10% heat-inactivated fetal calf serum, glutamine (2 mM), and antibiotics (penicillin and streptomycin).

Peripheral blood mononuclear cells were cultured (1 × 10^6^ cells/mL) in 96-well flat bottom plates and stimulated with phorbol myristate acetate (PMA; 20 ng/mL) and ionomycin (1µg/mL) (both from Sigma-Aldrich) in the presence of Brefeldin A (1µg/mL) (Sigma-Aldrich). Cultures were maintained 18 h at 37°C in a 5% CO_2_ atmosphere.

CD4^+^CXCR5^−^CD25^−^CD127^+^ effector T cells (Teff), CD4^+^CXCR5^−^CD25^high^CD127^low^ non follicular regulatory T cells (Treg), and CD4^+^CXCR5^+^CD25^high^CD127^low^ follicular regulatory T cells (Tfr) were sorted from PBMCs using a FACSAria Fusion sorter cytometer (Becton Dickinson) and resuspended in culture medium supplemented with 0.01 mM β2-mercaptoethanol (Fluka BioChemika). Sorted Teff cells were labeled during 5 min at RT (25°C) with 1 µg/mL CFSE (Invitrogen) following manufacturer’s instructions. 5 × 10^4^ CFSE-labeled Teff cells per well were cultured 4 days in 96-well round bottom plates coated overnight at 4°C with anti-CD3 1 µg/mL (UCHT1 clone; eBioscience) in phosphate-buffered saline (PBS). To evaluate the inhibition of Teff proliferation, unlabeled Treg and Tfr were added to the culture to a final volume of 150 μl/well. A CFSE dilution protocol was used to evaluate Teff cell proliferation.

### Flow Cytometry

Cell surface marker expression and intracellular cytokines were analyzed by flow cytometry using an Epics FC500 and Navios flow cytometers (Beckman Coulter). Data evaluation was done with the Kaluza software (Beckman Coulter).

A surface staining protocol was performed to analyze membrane antigen expression in B cells and cTfh cells. Briefly, 100 µL of peripheral whole blood were incubated with different combinations of fluorochrome-conjugated monoclonal antibodies 20 min at RT (25°C). Red blood cells were lysed and white cells fixed using TQ-Prep System (Coulter Corp.) before flow cytometry analysis. To evaluate B cells subpopulations and phenotypically classify CVID patients, combinations of the following antibodies were used: anti-CD19-ECD, anti-CD27-PCy7 (both from Coulter Immunotech), anti-IgD-FITC (Dako), anti-CD21-FITC (Coulter Immunotech), and anti-CXCR5-PE (R&D Systems). Different mixtures of anti-CD4-PCy5, anti-CD45RA-ECD, anti-PD1-PCy7, anti-CD127-FITC, anti-CD25-PCy5 (all from Coulter Immunotech), anti-CXCR5-PE (R&D Systems), anti-CXCR3-FITC, and anti-CCR6-PCy7 (GrupoTaper) antibodies were used to evaluate cTfh cells and their subpopulations.

An intracellular staining protocol was used to evaluate stimulation-induced cytokines expression in cultured cells following manufacturer’s instructions (IntraPrep Permeabilization Reagent from Beckman Coulter). Briefly, 2 × 10^4^ cultured cells were harvested, stained 15 min at RT in the dark with a combination of anti-CXCR5-PE (R&D Systems), anti-CD4-PCy7, and anti-CD3-ECD (both from Coulter Immunotech) or, alternatively, with anti-CXCR5-PE (R&D Systems), anti-CD3-PCy7, and anti-CD4-PCy5 (both from Coulter Immunotech). After surface staining, cells were washed with cold PBS, fixed with formaldehyde solution 15 min at RT in the dark, washed with cold PBS, and permeabilized with a saponine solution 20 min at RT in the dark. Intracellular staining was performed adding anti-INFγ-FITC (Coulter Immunotech) or anti-IL-17-Alexa 647 (BD Pharmingen) within the last 15 min of the permeabilization step. Finally, cells were washed, resuspended in PBS, and analyzed.

Foxp3 expression on PBMC and sorted T cells was evaluated by intracellular staining with a PE-conjugated mAb to Foxp3 (Becton Dickinson) following the fixation/permeabilization set manufacturer’s instructions (eBioscience). Unspecific intracellular staining was tested with a PE-labeled mouse IgG1 mAb (R&D Systems) as isotype control. Anti-CD127-FITC, anti-CD25-PCy5, and anti-CD4-PCy7 (all from Coulter Immunotech) were used as surface markers to identify the selected population, as described previously.

### Statistical Analysis

Statistical analysis was performed using GraphPad Prism version 4.0 software. Data are expressed as mean values. The Mann–Whitney *U*-test was used to compare differences between CVID patients and controls. The Kruskal–Wallis test was used to compare differences between subgroups of CVID patients and controls. A *p*-Value less than 0.05 was considered statistically significant.

## Results

### Circulating Tfh Cells Are Increased in CVID Patients

**Figure 1:**
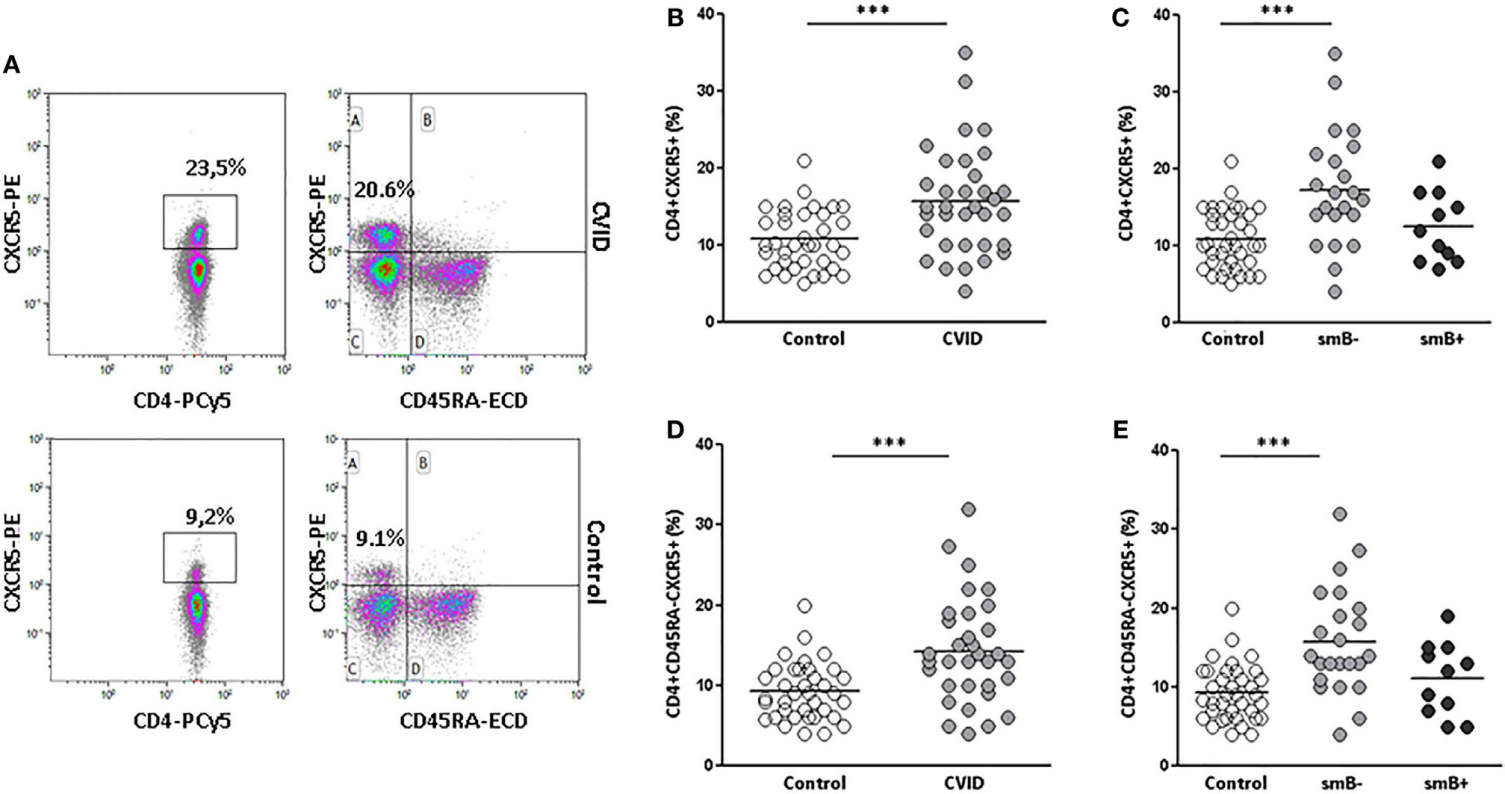
**Percentages of CD4^+^CXCR5^+^ and CD4^+^CD45RA^−^CXCR5^+^ circulating(c) follicular helper T cells are higher in peripheral blood samples from common variable immunodeficiency (CVID) patients than controls**. **(A)** Density plots of the percentage of CD4^+^CXCR5^+^ (left) and CD4^+^CD45RA^−^CXCR5^+^ (right) cells from a representative CVID patient (upper row) and control (lower row). **(B)** Differences (*p* < 0.001) between controls (open circles) and CVID patients (light gray circles). **(C)** Differences (*p* < 0.001) between controls (open circles) and smB^−^ CVID patients (light gray circles). No differences were found between both groups of patients or between smB^+^ CVID patients (dark gray circles) and controls. **(D)** Differences (*p* < 0.001) between controls (open circles) and CVID patients (light gray circles). **(E)** Differences (*p* < 0.001) between controls (open circles) and smB^−^ CVID patients (light gray circles). No differences were found between both groups of patients or between smB^+^ CVID patients (dark gray circles) and controls.

cTfh cells are identified in peripheral blood as CD4 T cells that co-express CXCR5 (28) (Figure 1A). We found a higher percentage of CD4^+^CXCR5^+^ T cells in CVID patients when compared to controls (15.71 vs. 10.79%; *p* < 0.001) (Figure 1B). When smB^−^ and smB^+^ patients were evaluated separately, differences were found between smB^−^ patients and controls (17.23 vs. 10.79%; *p* < 0.001), but neither between both groups of patients nor between smB^+^ patients and controls (Figure 1C). Three patients were studied at different time points and the percentages of CD4^+^CXCR5^+^ T cells were similar (patient 32 initial value: 9.1%; after 24 months: 9.9%; patient 8 initial value: 33%; after 27 months: 29%; patient 25 initial value: 16.7%; after 5 months: 16.8%).

Several studies have shown that cTfh cells are contained within the memory CD45RA^−^CD4^+^ T cells (28) (Figure 1A). For this reason, we also evaluated the percentage of circulating CD4^+^CD45RA^−^CXCR5^+^ cells in CVID patients and controls. We confirmed a higher percentage of CD4^+^CD45RA^−^CXCR5^+^ in CVID patients when compared to controls (14.24 vs. 9.24%; *p* < 0.001) (Figure 1D). These differences were higher when we compared smB^−^ patients and controls (15.75 vs. 9.24%; *p* < 0.001). Again, differences were restricted to the smB^−^ group (Figure 1E).

No differences were found between controls and smB^−^ or smB^+^ CVID patients in the distribution of naïve (CCR7^+^CD45RA^+^), central (CCR7^+^CD45RA^−^), and effector (CCR7^−^CD45RA^−^) memory subpopulations on circulating CD4^+^CXCR5^+^ cells (Table S1 in Supplementary Material).

### PD-1 Expression Is Increased in cTfh and Non-Follicular CD4 T Cells from smB^−^ CVID Patients

**Figure 2:**
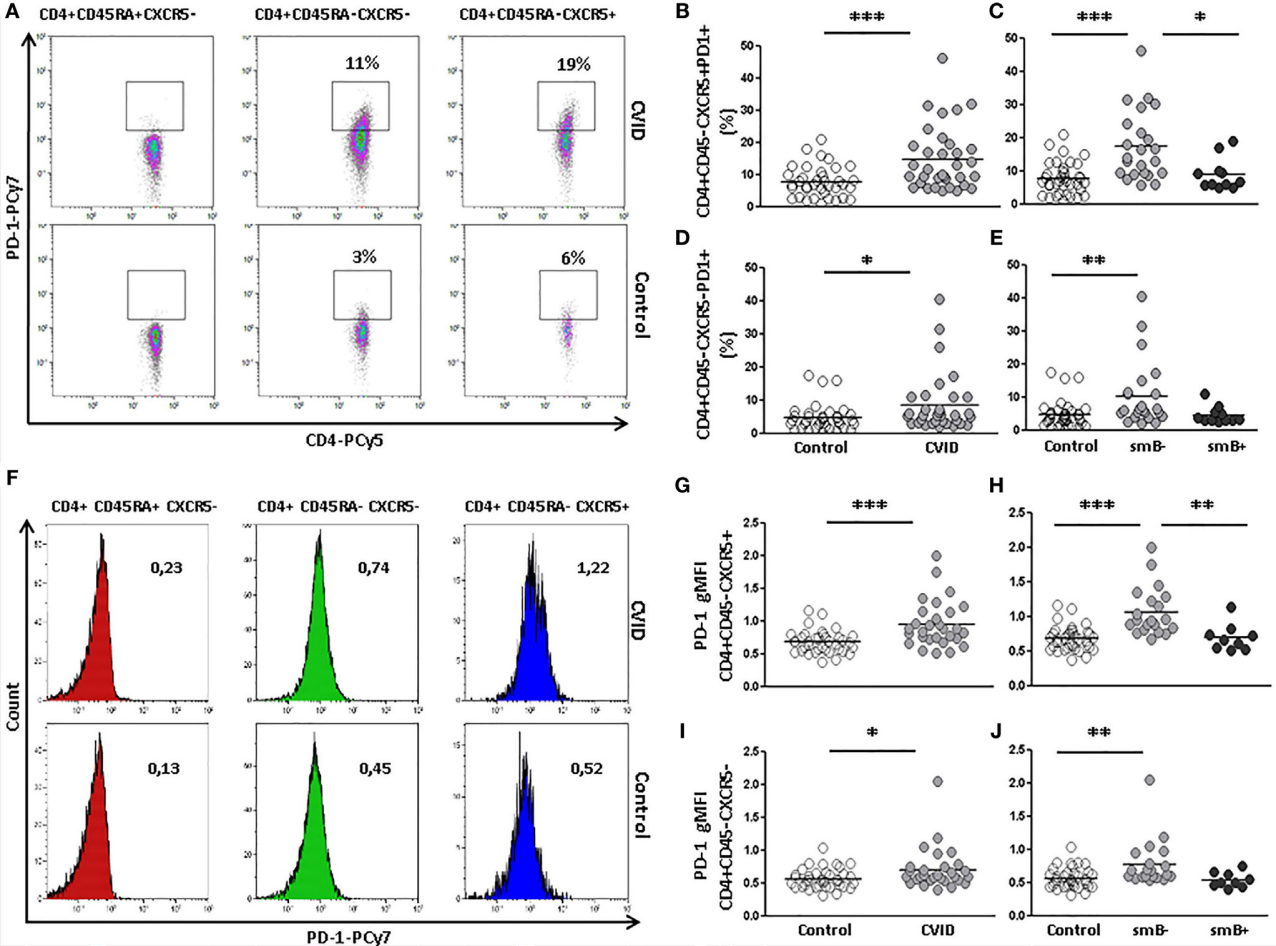
**Programmed death (PD)-1 expression on circulating CD4^+^CD45RA^−^CXCR5^+^ and CD4^+^CD45RA^−^CXCR5^−^ cells is higher in peripheral blood samples from CVID patients compared to controls**. **(A)** Density plots of percentage of PD-1 expression on CD4^+^CD45RA^+^CXCR5^−^ (left), CD4^+^CD45RA^−^CXCR5^−^ (middle), and CD4^+^CD45RA^−^CXCR5^+^ (right) cells from a representative CVID patient (upper row) and control (lower row). **(B)** Differences (*p* < 0.001) between controls (open circles) and CVID patients (light gray circles). **(C)** Differences (*p* < 0.001) between smB^−^ CVID patients (light gray circles) and controls (open circles) or smB^+^ CVID (dark gray circles) (*p* < 0.05). No differences were found between smB^+^ CVID patients and controls. **(D)** Differences (*p* < 0.05) between controls (open circles) and CVID patients (light gray circles). **(E)** Differences (*p* < 0.01) between controls (open circles) and smB^−^ CVID patients (light gray circles). No differences were found between both groups of patients or between smB^+^ CVID patients (dark gray circles) and controls. **(F)** Histograms of PD-1 gMFI on CD4^+^CD45RA^+^CXCR5^−^ (left), CD4^+^CD45RA^−^CXCR5^−^ (middle), and CD4^+^CD45RA^−^CXCR5^+^ (right) cells from a representative CVID patient (upper row) and control (lower row). **(G–J)** Differences of PD-1 gMFI expression corroborate differences found in percentages of PD-1 positive cells **(B–E)**.

Since Tfh cells are characterized by a variable expression of PD-1, we also evaluated the presence of PD-1 on circulating follicular CD4^+^CD45RA^−^CXCR5^+^ and non-follicular CD4^+^CD45RA^−^CXCR5^−^ T cells from CVID patients and controls (Figure 2A).

We found a higher percentage of PD-1-expressing cTfh cells in CVID patients compared to controls (14.71 vs. 7.69%; *p* < 0.001) (Figure 2B). These differences were also found when the expression of PD-1 was compared between smB^−^ CVID patients and controls (17.46 vs. 7.69%; *p* < 0.001) or smB^+^ CVID patients (17.46 vs. 8.97%; *p* < 0.05). We did not find differences between smB^+^ CVID patients and controls (Figure 2C). The expression of PD-1 on circulating CD4^+^CD45RA^−^CXCR5^+^ T from three CVID patients was similar when evaluated at different time points (patient 8 initial value: 9.9%; after 24 months: 7.3%; patient 32 initial value: 23.9%; after 27 months: 24.8%; patient 25 initial value: 8.7%; after 5 months: 10.0%).

The percentage of PD-1-expressing circulating non-follicular CD4^+^CD45RA^−^CXCR5^−^ T cells was also higher in CVID patients compared to controls (8.34 vs. 4.58%; *p* < 0.05) (Figure 2D). Differences were also found between smB^−^ CVID patients and controls (10.18 vs. 4.58%; *p* < 0.01), but not between both groups of CVID patients or between smB^+^ CVID patients and controls (Figure 2E).

The intensity of expression of PD-1 was also evaluated (Figure 2F). We found a higher intensity of PD-1 expression (gMFI) on cTfh cells from CVID patients compared to controls (0.95 vs. 0.67 gMFI; *p* < 0.001) (Figure 2G). These differences were also found when the levels of PD-1 were compared between smB^−^ CVID patients and controls (1.06 vs. 0.67 gMFI; *p* < 0.001) or smB^+^ CVID patients (1.06 vs. 0.69 *p* < 0.01). We did not find differences between smB^+^ CVID patients and controls (Figure 2H).

The expression of PD-1 was also higher on circulating non-follicular CD4^+^CD45RA^−^CXCR5^−^ T cells from CVID patients compared to controls (0.70 vs. 0.56 gMFI; *p* < 0.05) (Figure 2I). Differences were also found between smB^−^ CVID patients and controls (0.78 vs. 0.56 gMFI; *p* < 0.01), but not between both groups of CVID patients or between smB^+^ CVID patients and controls (Figure 2J).

### Increase of cTfh1 and Decrease of cTfh17 Effector Cells in smB^−^ CVID Patients

**Figure 3:**
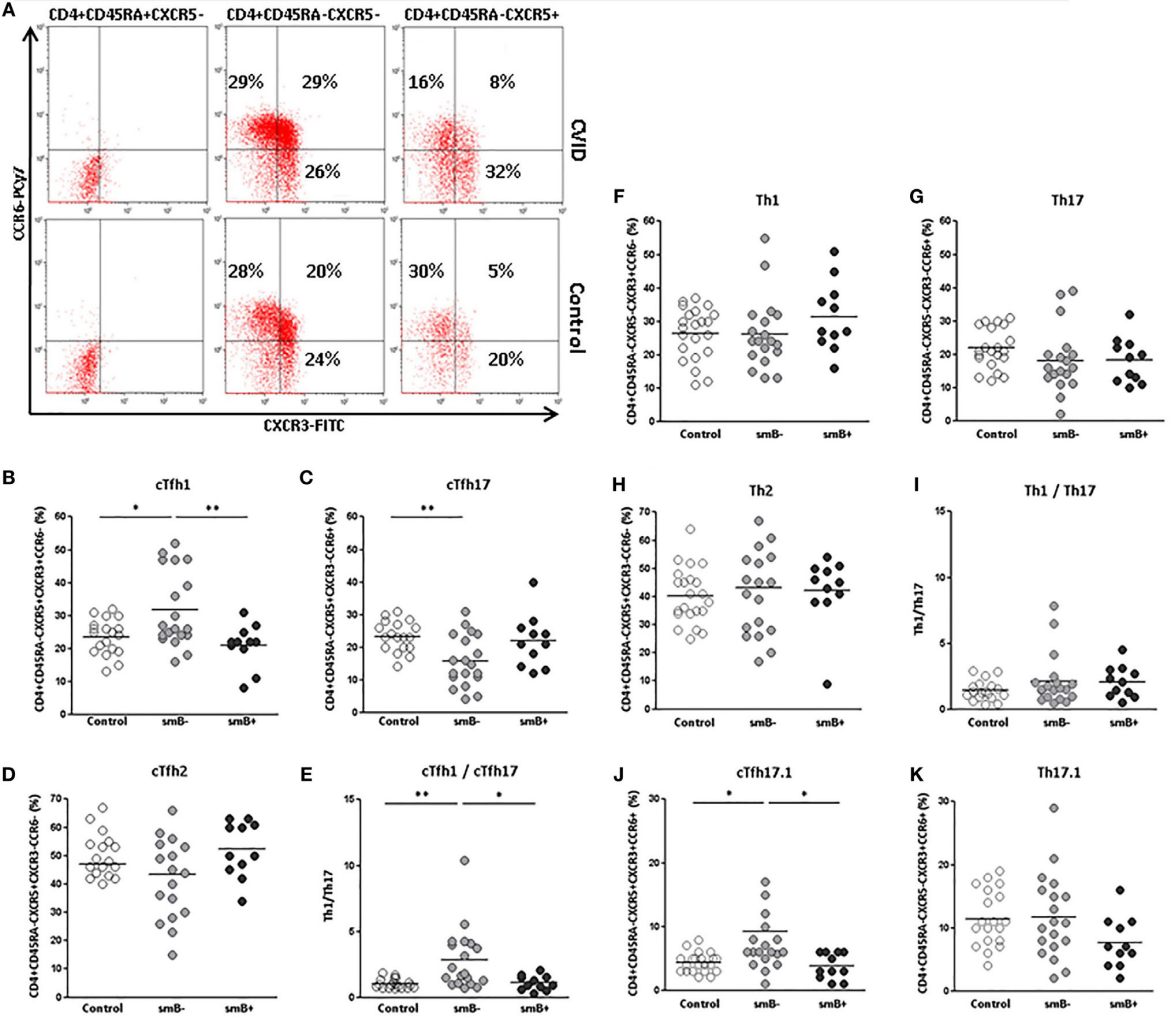
**Distribution of effector Th1 (CXCR3^+^CCR6^−^), Th2 (CXCR3^−^CCR6^−^), Th17 (CXCR3^−^CCR6^+^), and Th17.1 (CXCR3^+^CCR6^+^) subpopulations on circulating CD4^+^CD45RA^−^CXCR5^+^ and CD4^+^CD45RA^−^CXCR5^−^ cells in peripheral blood samples from CVID patients and controls**. **(A)** Dot-plots of the percentage of CXCR3^+^CCR6^−^ (lower right quadrant), CXCR3^−^CCR6^−^ (lower left quadrant), CXCR3^−^CCR6^+^ (upper left quadrant), and CXCR3^+^CCR6^+^ (upper right quadrant) subpopulations on circulating CD4^+^CD45RA^+^CXCR5^−^ (left), CD4^+^CD45RA^−^CXCR5^−^ (middle), and CD4^+^CD45RA^−^CXCR5^+^ (right) cells from a representative CVID patient (upper row) and control (lower row). **(B)** Significantly higher percentage of CXCR3^+^CCR6^−^ in cTfh cells from smB^−^ CVID patients (*p* < 0.05) (light gray circles) compared to controls (open circles) and smB^+^ CVID patients (*p* < 0.01) (dark gray circles). **(C)** Significantly lower percentage (*p* < 0.01) of CXCR3^−^CCR6^+^ of cTfh cells from smB^−^ CVID patients (light gray circles) compared to controls (open circles). No differences were found between smB^+^ CVID patients (dark gray circles) and controls or between both groups of patients. **(D)** No differences were found in the percentage of CXCR3^−^CCR6^−^ cTfh cells between smB^−^ (light gray circles) and smB^+^ (dark gray circles) or controls (open circles). **(E)** Higher cTfh1/cTfh17 ratio in smB^−^ CVID patients (light gray circles) compared to controls (open circles) or to smB^+^ CVID patients (dark gray circles). No differences were found between controls (open circles) and smB^−^ (light gray circles) or smB^+^ (dark gray circles) CVID patients, in the distribution of effector CXCR3^+^CCR6^−^ **(F)**, CXCR3^−^CCR6^+^ **(G)**, and CXCR3^−^CCR6^−^ **(H)** circulating non-follicular Th subpopulations, nor in the Th1/Th17 ratio **(I)**. **(J)** Significantly higher percentage (*p* < 0.05) of CXCR3^+^CCR6^+^ in cTfh cells from smB^−^ CVID patients (light gray circles) compared to controls (open circles) and smB^+^ CVID patients (*p* < 0.05) (dark gray circles). No differences were found between controls and smB^−^ or smB^+^ CVID patients, in the distribution of effector CXCR3^+^CCR6^+^ circulating non-follicular Th cells **(K)**.

Different subsets of effector circulating CD4^+^CD45RA^−^CXCR5^+^ T cells exist that are characterized according to the expression of the chemokine receptors CXCR3 and CCR6: cTfh1 (CXCR3^+^CCR6^−^), cTfh2 (CXCR3^−^CCR6^−^), and cTfh17 (CXCR3^−^CCR6^+^) (Figure 3A). We evaluated if there were differences in the distribution of effector subpopulations in cTfh and non-follicular CD4^+^ cells from CVID patients and controls.

We found a higher percentage of cTfh1 cells in smB^−^ CVID patients when compared to controls (31.69 vs. 23.40%; *p* < 0.05) (Figure 3B) and to smB^+^ CVID patients (31.69 vs. 21.00%; *p* < 0.01) (Figure 3B), whereas the percentage of cTfh17 cells was significantly decreased in cTfh cells from smB^−^ CVID patients when compared to controls (15.78 vs. 23.24%; *p* < 0.01) (Figure 3C). No differences were found when cTfh2 subpopulations were compared (Figure 3D). The cTfh1/cTfh17 ratio was significantly higher in smB^−^ CVID patients when compared to controls (2.84 vs. 1.05; *p* < 0.01) or smB^+^ CVID patients (2.84 vs. 1.10; *p* < 0.05) (Figure 3E). Interestingly, those patients with the highest percentage of cTfh1 cells (patients 6, 19, 20, 22, and 23), all of them smB^−^ CVID patients, had the lowest immunoglobulin levels at diagnosis (IgG < 40 mg/dl) (Table 1). However, these findings did not correlate with a higher incidence of autoimmunity and/or enteropathy (Table 1).

When effector Th1, Th17, and Th2 non-follicular CD4^+^CD45RA^−^CXCR5^−^ T cells subpopulations were evaluated, no differences were found between CVID patients and controls (Figures 3F–I).

A CXCR3^+^CCR6^+^ Th subpopulation that rivals with Th1 in INFγ production has been recently described as Th17.1 and identified in sarcoidosis and Crohn’s disease (29–32). We found an increase in the percentage of an analogous CXCR3^+^CCR6^+^ expressing Tfh population in the smB^−^ group compared to controls (9.25 vs. 4.36%; *p* < 0.05) and to smB^+^ CVID patients (9.25 vs. 3.81%; *p* < 0.05), but not between smB^+^ CVID patients and controls (Figures 3A,J). No differences were found in Th17.1 non-follicular CD4^+^CD45RA^−^CXCR5^−^ T cells percentages between CVID patients and controls (Figure 3K).

These parameters were evaluated again after 5 months in some patients and the percentages of the different subsets of effector circulating CD4^+^CD45RA^−^CXCR5^+^ T cells (cTfh1, cTfh2, and cTfh17) remained similar (data not shown).

### INFγ and IL-17 Expression in cTfh Cells from CVID Patients and Controls

We studied if the alterations observed in the distribution of different effector cTfh cell subpopulations translated into the production of signature cytokines of each population. We evaluated the percentage of IFNγ or IL-17-producing cTfh and non-follicular CD4 T after stimulation of PBMC with PMA and ionomycin.

**Figure 4:**
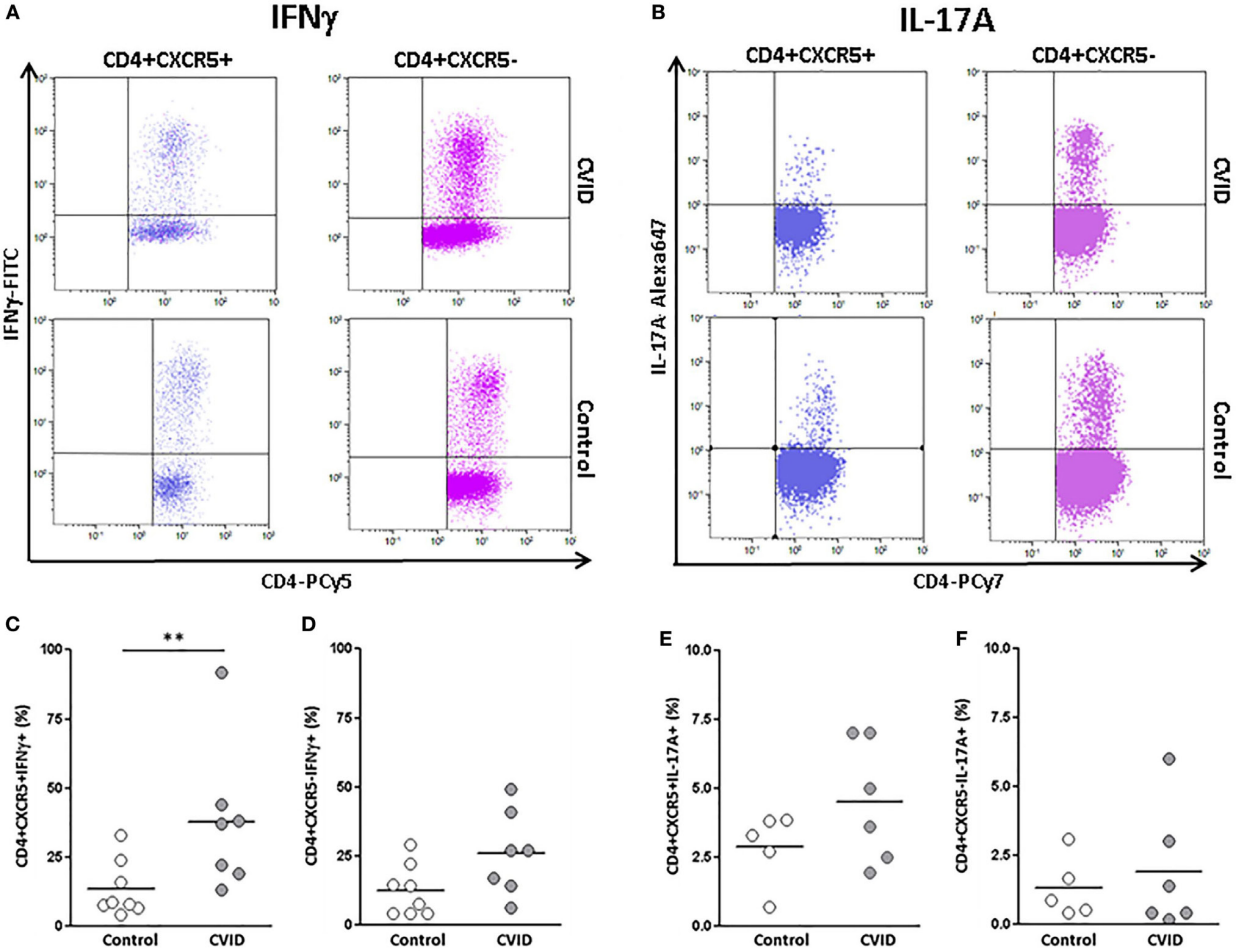
**Percentages of INFγ and interleukin (IL)-17A-producing CD4^+^CXCR5^+^ and CD4^+^CXCR5^−^ cells in peripheral blood samples from CVID patients and controls**. **(A)** Dot-plots of the percentage of INFγ^−^producing CD4^+^CXCR5^+^ (left) and CD4^+^CXCR5^−^ (right) cells from a representative CVID patient (upper row) and control (lower row). **(B)** Dot-plots of the percentage of IL-17A-producing CD4^+^CXCR5^+^ (left) and CD4^+^CXCR5^−^ (right) cells from a representative CVID patient (upper row) and control (lower row). **(C)** Significantly higher percentage (*p* < 0.01) of INFγ^−^producing CD4^+^CXCR5^+^ cells in CVID patients (light gray circles) compared to controls (open circles). No differences were found in INFγ producing CD4^+^CXCR5^−^ cells between CVID patients (light gray circles) and controls (open circles) **(D)**. No differences were found in the percentage of IL-17A-producing CD4^+^CXCR5^+^ **(E)** or CD4^+^CXCR5^−^ **(F)** cells between CVID patients (light gray circles) and controls (open circles).

We found a higher percentage of IFNγ^−^producing cTfh in CVID patients than in controls (37.00 vs. 13.95%; *p* < 0.01). IFNγ^−^producing non-follicular CD4^+^CXCR5^−^ T cells were also increased in CVID patients in comparison to controls, although in this case differences did not reach statistical significance (26.25 vs. 12.92%; *p* = 0.053) (Figures 4A,C,D). No differences were found in IL-17 production between CVID patients and controls, neither in cTfh nor in non-follicular CD4^+^CXCR5^−^ (Figures 4B,E,F). Therefore, CD4^+^CXCR5^+^ and CD4^+^CXCR5^−^ producing INFγ subpopulations were increased in CVID patients in agreement with the previously observed phenotypic results.

### Follicular Regulatory T Cells and Regulatory T Cells Are Decreased in smB^−^ CVID Patients

**Figure 5:**
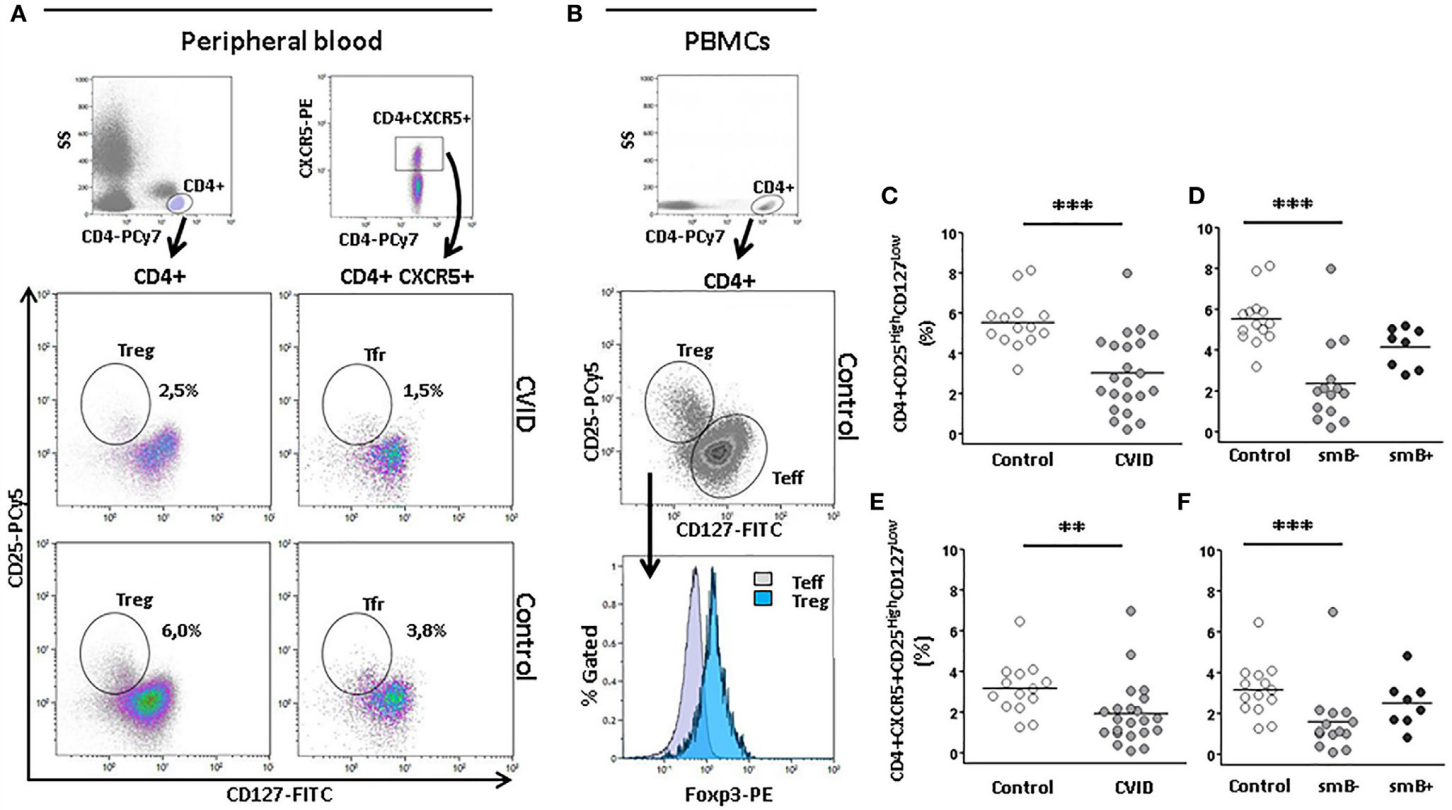
**Percentage of CD4^+^ CD25^high^CD127^low^ (Treg) and CD4+CXCR5^+^CD25^high^CD127^low^ regulatory follicular T(Tfr) cells are lower in peripheral blood from CVID patients than controls**. **(A)** Gating strategy of Treg (CD4^+^CD25^high^CD127^low^) and Tfr (CD4^+^CXCR5^+^CD25^high^CD127^low^) and density plots from a representative CVID patient (upper row) and a healthy control (lower row). Panel **(B)** shows that Foxp3 positive cells are contained within the CD25^high^CD127^low^ CD4 cells. **(C)** Differences in percentage of Tregs between CVID patients (light gray circles) and controls (open circles) (*p* < 0.001) and **(D)** between smB^−^ CVID patients (light gray circles) and controls (open circles) (*p* < 0.001). No differences were found between smB^+^ CVID patients (dark gray circles) and controls or between both groups of patients. **(E)** Differences in percentage of Tfr between CVID patients (light gray circles) and controls (open circles) (*p* < 0.01) and **(F)** between smB^−^ CVID patients (light gray circles) and controls (open circles) (*p* < 0.001). No differences were found between smB^+^ CVID patients (closed circles) and controls or between both groups of patients.

We identified regulatory T (Treg) cells by flow cytometry as CD4^+^CD25^high^CD127^low^ T cells and follicular regulatory T cells (Tfr) as CD4^+^CXCR5^+^CD25^high^CD127^low^ T cells, respectively, as previously described (Figure 5A) (33). Foxp3-expressing cells are contained within the CD25^high^CD127^low^ subpopulation of CD4^+^ cells (Figure 5B).

Several studies have shown that Treg are reduced in CVID (34). This regulatory T cell subpopulation was significantly decreased in our group of CVID patients compared to controls (3.00 vs. 5.50%; *p* < 0.001) (Figure 5C). The frequency of Treg cells was also reduced in smB^−^ patients compared to controls (2.40 vs. 5.50%; *p* < 0.001), but not to smB^+^ patients. No differences were found between smB^+^ patients and controls (Figure 5D).

Moreover, we studied the Tfr cells, and we found a strikingly lower percentage of this subpopulation in CVID compared to controls (1.90 vs. 3.20%; *p* < 0.01) (Figure 5E). The percentage Tfr cells was also significantly decreased in smB^−^ patients compared to controls (1.60 vs. 3.20%; *p* < 0.001), but not to smB^+^ patients. No differences were found between smB^+^ patients and controls (Figure 5F).

**Figure 6:**
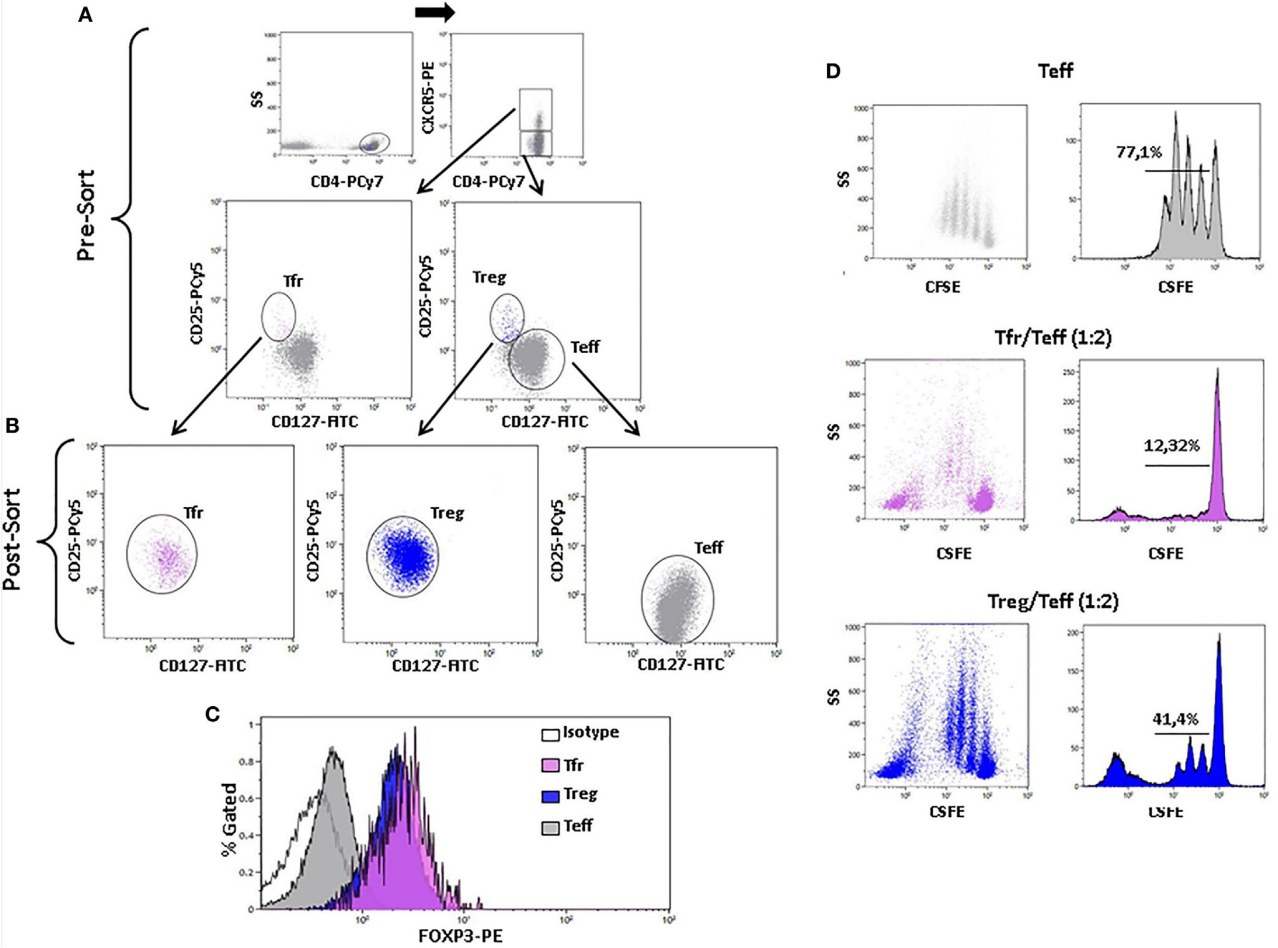
**Foxp3 expression and inhibitory function of sorted CD4^+^CXCR5^+^CD25^high^CD127^low^ regulatory follicular T (Tfr) and non-follicular CD4^+^CXCR5^−^CD25^high^CD127^low^ (Treg) cells**. **(A)** Sorting strategy of Tfr and non-follicular Treg cells. **(B)** CD25 and CD127 expression of sorted Tfr (post-sort left, pink), Treg (post-sort center, blue), and CD4^+^CXCR5^−^CD25^−^CD127^+^ T effector (Teff) (post-sort right, gray) cells. **(C)** Histogram of Foxp3 expression of Tfr (pink), Treg (blue), and Teff (gray) cells. **(D)** Dot plots (left column) and histograms (right column) showing proliferation of Teff alone (upper row) or in the presence of Tfr at a 1:2 Tfr/Teff (middle row) or Treg at a 1:2 Treg/Teff (lower row) ratio.

To confirm that Tfr cells were indeed regulatory T cells, we sorted Tfr and non-follicular Treg (Figures 6A,B) and evaluated their intracellular expression of Foxp3. Both sorted non-follicular Treg and Tfr expressed Foxp3 in contrast to sorted Teff cells (Figure 6C). To study their regulatory function, we evaluated the inhibition of proliferation of sorted Teff cells cultured with non-follicular Treg or Tfr (ratio Treg or Tfr/Teff 0:1 and 1:2). Figure 6D shows that both subpopulations inhibit proliferation of Teff cells. These results demonstrate that CD4^+^CXCR5^+^CD25^high^CD127^low^ T cells express Foxp3^+^ and exert regulatory function as their non-follicular CD4^+^CXCR5^−^CD25^high^CD127^low^ counterparts, which validate the gating strategy.

## Discussion

In the present study, we have found an increase in the percentage of cTfh (CD4^+^CXCR5^+^) cells in CVID patients, especially in those with a more compromised memory B cell compartment (smB^−^ group). These cells express higher levels of PD-1 and are skewed toward a Th1 phenotype: cTfh1 (CXCR3^+^CCR6^−^) and a putative cTfh17.1 (CXCR3^+^CCR6^+^) are increased whereas cTfh17 (CXCR3^−^CCR6^+^) are decreased. No differences were found in the cTfh2 (CXCR3^−^CCR6^−^) subpopulation. Moreover, CVID cTfh cells also produce higher levels of INFγ. cTfr cells were strikingly decreased in CVID patients as were Treg cells.

Follicular helper T cells are essential for the germinal center reaction (35) and the generation of high affinity, long-lived antibody responses (36, 37). Studies of Tfh have been dampened in humans due to their location in secondary lymphoid organs. The cTfh population was identified as a surrogate memory counterpart of lymphoid Tfh cells in peripheral blood (14). These cells are CXCR5^+^, but they express variable levels of other characteristic markers of Tfh populations like PD-1 or ICOS. Moreover, functional heterogeneity exists among cTfh subpopulations: cTfh1, cTfh17, and cTfh2 (analogous to Th1, Th2, and Th17 cells) can be distinguished according to CXCR3 and CCR6 chemokine receptor expression. cTfh2 and cTfh17 cells are able to help naïve B cells to produce immunoglobulins *via* IL-21 whereas cTfh1 cells lack the capacity to help B cells (14).

Alterations in cTfh have been extensively associated with autoimmune disease, infectious disease, and more recently, with immunodeficiency. Increases of cTfh have been described in patients with systemic lupus erythematosus (19), rheumatoid arthritis (38), dermatomyositis (14), autoimmune thyroid disease (39), Sjögren’s syndrome (40), myasthenia gravis (41), multiple sclerosis (21), and other autoimmune diseases.

On the contrary, low frequencies of CD4^+^CXCR5^+^ cells have been described in several monogenic PIDs like ICOS deficiency (22), X-linked agammaglobulinaemia (23), STAT3 deficiency (24), and also IL10R, CD40L, and NEMO mutations (25). However, other studied PIDs with monogenic mutations like IFNGR1/2 deficiency, IL-21 or IL-21R deficiency, GOF STAT1, and LOF STAT1, SH2D1A, did not show any difference in the frequency of CD4^+^CXCR5^+^ cells (25).

Strikingly, we have found an increase of CD4^+^CXCR5^+^ cells in CVID patients. When patients were separated into smB^−^ and smB^+^ patients, the difference was significant only for the smB^−^ group indicating a relationship between the altered cTfh cells and the “compromised memory B cells generation.” Moreover, CD4^+^CXCR5^+^ cells from our smB^−^ patients express higher levels of PD-1 than normal controls. Initially identified as a molecule responsible for induction of cell death, PD-1 is now considered a dominant inhibitor of T cell effector responses important in the regulation of humoral immune responses (42–45). The increased PD-1 expression in cTfh cells from smB^−^ patients may contribute to functional changes in this subpopulation.

Mutations in PIDs can affect not only quantity but also quality of Tfh cells decreasing their B cell cooperation ability (25). We have found a skewing toward cTfh1 phenotype in our patients, with increased production of INFγ and a decrease in cTfh17 cells especially in the smB^−^ group. Our data are in agreement with previous studies published by Ma et al. who found a cTfh1 skewing in several monogenic PIDs like LOF STAT3 and GOF STAT1 mutations, with a significant increase of CXCR3^+^ subpopulation and a consistent decrease of CCR6^+^ subpopulation, mirrored by a corresponding skewing of cytokines. Patients with these mutations present a defect in memory B cells maturation, among other characteristics, as do smB^−^ CVID patients. Moreover, INFγ was mostly secreted by purified CXCR3^+^ and CXCR3^+^CCR6^+^ cTfh subpopulations, IL-17A/F and IL-22 were secreted mainly by CCR6^+^ cTfh cells, whereas all subpopulations were able to differentiate to IL-21-producing cells. They also demonstrated that CCR6^+^ cells, diminished in our smB^−^ CVID patients, were the most potent inductors of immunoglobulin production by co-cultured B cells. Thus, these mutations not only compromise the generation of cTfh subsets, preventing the differentiation of the most potent B cell helper cells (25).

Our results are also consistent with the fact that, although the presence of cTfh cells in humans correlate with antibody responses to influenza vaccination (46) and production of neutralizing antibodies in HIV (47), Cubas et al. (48) found that, even if present at normal numbers, cTfh in a subgroup of chronic aviremic HIV-infected patients, did not cooperate properly with autologous memory B cells in co-culture. Memory B cells from these patients produced less IgG and specific antibodies when co-cultured with autologous cTfh cells. This was apparently due to dysfunctional characteristics of the cTfh cells that showed a polarized Th1 phenotype and produced increased amounts of INFγ that negatively correlated with the production of IgG. In keeping with this, those smB^−^ CVID patients in our cohort with the highest percentage of cTfh1 cells had the lowest immunoglobulin levels at diagnosis.

The Tfh1 skewing in our CVID patients was maintained over time and not related to higher incidence of autoimmunity and/or enteropathy in the smB^−^ group compared to the smB^+^ group. In one of the patients (patient 23), the skewing was present at the time of diagnosis, before treatment with gammaglobulin was initiated, supporting the hypothesis that cTfh1 cells increase could play a role in CVID onset, rather than being a consequence of the disease progression.

Obeng-Adjei et al. found that the inefficient acquisition of humoral responses to malaria in children is neither due to a deficiency in the generation and maintenance of memory Tfh cells nor to an altered distribution of Tfh cell subsets, but to the preferential activation of a Th1 polarized CD4^+^PD1^+^CXCR3^+^CXCR5^+^ subpopulation during acute malaria infection. The increase in this peripheral blood subpopulation during natural infection does not correlate with the increase of plasma cells or the breadth or magnitude of *P. falciparum*-specific antibodies (49).

Apart from their skewed Tfh1 phenotype, cTfh cells from our smB^−^ patients show an increase in CXCR3^+^CCR6^+^ analogous to the recently described Th17.1 subpopulation. These helper effector cells express and produce high levels of INFγ and have been found increased in Crohn’s disease (29) and in the lungs of the granulomatous disease (GD) sarcoidosis (31). Autoimmune and GDs are a frequent finding in CVID patients. Although still not clear, several cytokines including IL-1β, IL-12, and IL-23 have been implicated in the differentiation of this population (50). Consistently, we have previously found an increase of IL-12 in the sera of CVID patients (51), which could be related to the increase of this subpopulation. Moreover, Cambronero et al. reported an elevated IL-12 production by LPS-stimulated CVID monocytes accompanied by a raise of INFγ^−^producing T cells (52). We do not find a decreased production of IL-17 by cTfh cells in spite of the reduction of cTfh17 cells. This may be due to the fact that the CXCR3^+^CCR6^+^ cTfh subpopulation, increased in our patients, also produces IL-17 besides INFγ (25).

A high frequency of Tfh PD1^+^ cells was reported by Coraglia et al. in a subgroup of eight CVID patients characterized by the presence of GD and/or autoimmunity if compared to controls or a CVID group without GD or autoimmunity (53). Martinez-Gallo et al. found a high frequency of Tfh PD1^+^ cells and low frequency and function of Treg in a subgroup of CVID patients, related to the presence of autoimmunity and autoreactive B cells (54). In keeping with previous studies (34), we have also found a decrease in the percentage of Tregs in smB^−^ CVID patients, but also and more important, a striking decrease in Tfr cells. However, we did not find differences in Treg or Tfr when patients were grouped according to the presence or absence of autoimmunity and/or enteropathy (data not shown). Tfr derive from Treg cells and are presumed to suppress germinal center reaction through their access to the follicles. Although they can inhibit both Tfh and B cells, their mechanism of action is complex and not completely understood. Tfr may suppress B cells at many stages of their differentiation, even plasma cells (55), although the repercussion in the generation of high affinity antibodies is still controversial. The decrease in Treg and Tfr might be directly or indirectly related to the failure to generate of a normal B cell compartment in the smB^−^ CVID group.

In summary, the combination of a skewed Tfh response and a decrease in the Tfr population may compromise the generation of a functional B cell compartment and efficient humoral response in smB^−^ CVID patients.

## Author Contributions

JF and JP conceived, designed, and supervised the study. VC and NL performed the experiments. CB and VA contributed to the execution of the experiments. JP recruited CVID patients and provided clinical data. JF, JP, VC, AC, and NL analyzed experimental data. JF, JP, VC, and AC wrote the manuscript.

## Conflict of Interest Statement

The authors declare that the research was conducted in the absence of any commercial or financial relationships that could be construed as a potential conflict of interest.
