# Supplementary material for: Follicular T Cells from smB− Common Variable Immunodeficiency Patients Are Skewed Toward a Th1 Phenotype
Source: Front Immunol. 2017 Feb 27;8:174. doi: 10.3389/fimmu.2017.00174 (PMC5326800; doi:10.3389/fimmu.2017.00174)
Supplement: Supplementary file 1 [file Table_1.DOCX]

**Supplementary Table 1.** Distribution of naïve, central memory and effector memory in non follicular and follicular CD4 T cells of CVID patients.

| **Patient** | **CD4+ (%)** | | | **CD4+ CXCR5- (%)** | | | **CD4+ CXCR5+ (%)** | | |
| --- | --- | --- | --- | --- | --- | --- | --- | --- | --- |
|  | **CCR7+**  **CD45RA+** | **CCR7+**  **CD45RA-** | **CCR7-CD45RA-** | **CCR7+**  **CD45RA+** | **CCR7+**  **CD45RA-** | **CCR7-CD45RA-** | **CCR7+**  **CD45RA+** | **CCR7+**  **CD45RA-** | **CCR7-CD45RA-** |
| 1 | 11 | 74 | 13 | 13 | 71 | 13 | 4 | 87 | 9 |
| 2 | 40 | 51 | 7 | 44 | 45 | 7 | 9 | 84 | 6 |
| 3 | 44 | 51 | 2 | 50 | 45 | 1 | 11 | 86 | 2 |
| 4 | 31 | 59 | 6 | 8 | 83 | 8 | 13 | 82 | 5 |
| 5 | 7 | 81 | 10 | 8 | 80 | 11 | 3 | 90 | 5 |
| 6 | 3 | 79 | 18 | 1 | 85 | 13 | 2 | 88 | 8 |
| 7 | 31 | 58 | 8 | 39 | 48 | 8 | 10 | 83 | 6 |
| 8 | 45 | 45 | 5 | 49 | 41 | 5 | 23 | 71 | 4 |
| 9 | 15 | 68 | 15 | 16 | 66 | 15 | 8 | 79 | 12 |
| 10 | 38 | 54 | 5 | 42 | 49 | 5 | 11 | 85 | 4 |
| 11 | 26 | 57 | 12 | 31 | 53 | 12 | 13 | 79 | 7 |
| 12 | 32 | 63 | 4 | 36 | 56 | 4 | 11 | 82 | 4 |
| 13 | 36 | 56 | 8 | 35 | 53 | 8 | 17 | 75 | 7 |
| 14 | 20 | 65 | 10 | 22 | 62 | 11 | 11 | 80 | 8 |
| 15 | 20 | 65 | 11 | 22 | 62 | 11 | 6 | 85 | 8 |
| 16 | 3 | 86 | 13 | 4 | 80 | 15 | 3 | 87 | 10 |
| 17 | 15 | 73 | 10 | 15 | 70 | 11 | 10 | 83 | 6 |
| 18 | 15 | 79 | 5 | 16 | 78 | 5 | 7 | 91 | 2 |
| 19 | 10 | 69 | 17 | 12 | 66 | 19 | 5 | 87 | 8 |
| 20 | 31 | 57 | 8 | 38 | 48 | 8 | 10 | 84 | 5 |
| 21 | 19 | 68 | 10 | 18 | 71 | 8 | 8 | 87 | 5 |
| 22 | 11 | 68 | 18 | 12 | 66 | 19 | 3 | 84 | 12 |
| 23 | NA | NA | NA | NA | NA | NA | NA | NA | NA |
| 24 | 33 | 62 | 5 | 38 | 54 | 5 | 9 | 87 | 4 |
| 25 | 31 | 62 | 5 | 36 | 57 | 5 | 10 | 85 | 4 |
| 26 | 55 | 40 | 3 | 59 | 36 | 3 | 19 | 77 | 3 |
| 27 | 51 | 41 | 6 | 62 | 31 | 3 | 8 | 85 | 6 |
| 28 | 54 | 33 | 8 | 56 | 30 | 8 | 16 | 72 | 1 |
| 29 | 30 | 57 | 8 | 33 | 54 | 9 | 14 | 82 | 3 |
| 30 | NA | NA | NA | NA | NA | NA | NA | NA | NA |
| 31 | 24 | 62 | 12 | 27 | 59 | 12 | 7 | 84 | 8 |
| 32 | 38 | 46 | 10 | 38 | 45 | 10 | 17 | 80 | 3 |
| 33 | 39 | 52 | 5 | 43 | 48 | 5 | 9 | 85 | 5 |
| 34 | NA | NA | NA | NA | NA | NA | NA | NA | NA |
| Normal  range* | 36±13.0 | 52±13.8 | 6.7±-3.0 | 39±13.0 | 50.5±13.0 | 6.8±3.0 | 10.7±4.9 | 82.8±5.7 | 5.6±3.3 |

Percentages of naïve (CCR7+CD45RA+), central memory (CCR7+CD45RA-) and effector memory (CCR7-CD45RA-) subpopulations referred to total circulating CD4+, non follicular CD4+CXCR5- and follicular CD4+CXCR5+ T cells of CVID patients. NA: not available. *Normal values were obtained from the healthy controls included in the study (mean±SD).
